# Contact Occupancy and Per-Residue Energetic Contributions Distinguish Aflatoxin B1 Nanobodies with Different Binding Affinities

**DOI:** 10.3390/biom16091265

**Published:** 2026-09-01

**Authors:** Hualian Mo, Wenxing Chen, Zhaoxi Yang, Junjie Zhou, Xinze Zhang, Siyuan Yang, Xiaoman She, Wenyang Zhang, Shijuan Yan, Shaowen Wu

**Affiliations:** 1 State Key Laboratory of Swine and Poultry Breeding Industry, Agro-Biological Gene Research Center, Guangdong Academy of Agricultural Sciences, Guangzhou 510640, China; 20243184049@stu.scau.edu.cn (H.M.); 1073324010094@st.gsau.edu.cn (W.C.); 2025023251@m.scnu.edu.cn (Z.Y.); 2230503263@stu.gdpu.edu.cn (J.Z.); 20242190072@stu.scau.edu.cn (X.Z.); 1073324010132@st.gsau.edu.cn (S.Y.); zhangwenyang@agrogene.ac.cn (W.Z.); 2 School of Life Sciences, South China Normal University, Guangzhou 510631, China; 3 Guangdong Provincial Key Laboratory of High Technology for Plant Protection, Institute of Plant Protection, Guangdong Academy of Agricultural Sciences, Guangzhou 510640, China; shexiaoman@gdppri.com

**Keywords:** aflatoxin B1, nanobody, molecular recognition, molecular dynamics simulation, interaction fingerprint

## Abstract

Aflatoxin B1 (AFB1) is a potent foodborne mycotoxin for which nanobodies offer compact, engineerable detection reagents, yet the molecular basis of their affinity differences remains poorly defined. Here, closely related nanobodies G8, NB28, and NB26 were compared. Fluorescence correlation spectroscopy showed G8 had the lowest dissociation constant for AFB1-BSA, followed by NB28 and NB26. To resolve this affinity separation, we performed triplicate 3 μs molecular dynamics simulations for each complex. While all complexes remained stable, G8 maintained broader, persistent aromatic and hydrophobic contacts extending into its elongated CDR3, whereas NB28 and NB26 relied on localized contacts centered on PHE47 and TRP101. Per-residue MM/GBSA decomposition confirmed favorable thermodynamic contributions from these persistent G8 residues. Ultimately, integrating contact occupancy, energy decomposition, and pairwise co-occurrence identified distinct cooperative contact networks (six in G8, versus two in NB28/NB26). This workflow demonstrates how mapping dynamic, cooperative binding networks rather than static proximity can systematically prioritize candidate residues for engineering high-affinity detection reagents in food-safety surveillance.

## 1. Introduction

Aflatoxin B1 (AFB1) is a naturally occurring mycotoxin of major concern to food safety because dietary exposure has been linked to hepatocellular carcinoma and other adverse health outcomes [1,2,3]. Its frequent occurrence in agricultural commodities has therefore motivated the development of analytical methods that combine high sensitivity with operational simplicity. Immunoassays remain important for this purpose, but their performance depends critically on the affinity, specificity, stability, and engineerability of the molecular recognition reagent [4].

Nanobodies, or variable domains of camelid heavy-chain-only antibodies (VHHs), provide an attractive binding scaffold because they are compact, stable, readily produced in microbial hosts, and amenable to sequence-level engineering [5,6,7]. These properties have enabled VHH-based assays for environmental chemicals and food contaminants, including AFB1 [8,9,10]. For small molecules, however, affinity cannot generally be inferred from a static sequence motif or a single contact residue. The ligand-binding surface comprises a compact arrangement of framework and complementarity-determining region (CDR) residues, and changes in its composition or spatial arrangement can alter binding. Structural studies of small-molecule-binding VHHs have accordingly shown that both CDRs and framework residues can contribute to ligand binding [11,12,13].

The AFB1 nanobodies NB26 and NB28 offer a well-defined precedent for this problem. Solution NMR and titration analyses localized the NB26 AFB1-binding interface primarily to CDR3 and framework region 2 (FR2); structure-guided substitutions at corresponding NB28 positions improved analytical sensitivity [14]. These findings establish the importance of defining the molecular basis of AFB1 binding, yet they do not explain why related nanobodies have different affinities or how multiple ligand-contacting residues contribute within the bound complex. G8, NB28, and NB26 provide an informative comparative set because they retain the VHH scaffold while differing across the CDRs, particularly in CDR3, and have been used as AFB1-binding reagents in nanobody-luciferase assays [15,16].

Here, we combined fluorescence correlation spectroscopy (FCS) affinity measurements with molecular dynamics (MD) simulations of AFB1-bound nanobody complexes. We established the experimental affinity order and then evaluated global stability, residue-resolved interaction fingerprints, representative complex conformations, mapped contact persistence, and MM/GBSA residue decomposition. Aromatic/hydrophobic contact occupancy was compared with per-residue decomposition, and frame-resolved pairwise contact co-occurrence was calculated to determine whether contacts occurred concurrently. Our results identify features of the bound complexes associated with stronger binding and guide the engineering of high-affinity detection reagents for food safety.

## 2. Materials and Methods

### 2.1. Materials and Reagents

AFB1 was purchased from Tan-Mo Technology Co., Ltd. (Changzhou, China). AFB1-BSA conjugates were obtained from Shenzhen Anti-Biological Technology Co., Ltd. (Shenzhen, China). Restriction enzymes were purchased from Thermo Fisher Scientific Co., Ltd. (Shanghai, China). Sulfo-Cyanine5 NHS ester purchased from Lumiprobe Limited Co., Ltd. (Shenzhen, China). All other reagents were of analytical or molecular biology grade and were purchased from standard commercial suppliers.

### 2.2. Plasmid Construction

The amino acid sequences of the AFB1-binding nanobodies G8, NB28, and NB26, and of NanoLuc luciferase (Nluc), were obtained from published studies [14,15]. Nluc was fused to G8, NB28, or NB26 through a GGSGGSGGSGGS linker. The corresponding coding sequences were synthesized by Sangon Biotech Co., Ltd. (Shanghai, China) and cloned into pET.M.3C through BamHI and EcoRI restriction sites. All constructs were verified by sequencing before transformation into *Escherichia coli* BL21(DE3) for recombinant protein expression.

### 2.3. Protein Expression and Purification

Expression vectors with verified sequences were introduced into *E. coli* BL21(DE3) cells, and protein expression and purification followed the established protocol. Briefly, single transformed colonies were cultured overnight at 37 °C, with shaking at 200 rpm. These cultures were then diluted 1:100 in fresh LB medium and cultivated to an approximate OD600 of 0.6–0.8. Induction with 0.3 mM IPTG followed, and the cultures were further incubated for 16–18 h at 16 °C before harvest. After centrifugation at 4000× rpm for 15 min at 4 °C, the pelleted cells were then resuspended in lysis buffer (20 mM Tris, 500 mM NaCl, 5 mM imidazole, pH 7.9), supplemented with 1 mM phenylmethanesulfonyl fluoride. Following ultrasonication and centrifugation, His-tagged proteins were purified from the lysates using immobilized nickel affinity chromatography, with elution performed using 1 M imidazole. Eluates were subsequently applied to a prepacked desalting column, with a chromatographic buffer of 50 mM phosphate buffer, pH 6.8, and their storage was maintained at −80 °C until use.

### 2.4. Fluorescence Correlation Spectroscopy (FCS)

Molecular hydrodynamic measurements were conducted using a bench-top FCS instrument (CorTector SX-B10; LightEdge Technologies Ltd., Zhongshan, China) equipped with dual continuous-wave lasers (532 nm and 638 nm) and a high-numerical-aperture Olympus 60X NA1.2 water immersion objective [17]. FCS measurements were performed using optimized conditions: 10 nM Cy5-labelled AFB1-BSAs were excited using the 638 nm laser, with each measurement lasting 10 s. Autocorrelation curves were analyzed using specialized Correlation Analysis software V3.7.011025 (LightEdge Technologies Limited) employing the following mathematical model:

$$G\left(\tau \right)\;=\;\frac{1}{N}\cdot \frac{\left(1\;-\;T\;+\;Te^{-\frac{\tau }{\tau_{T}}}\right)}{1\;-\;T}\cdot \frac{1}{(1\;+\;\frac{\tau }{\tau_{D}})\sqrt{1\;+\;\frac{\tau }{\tau_{D}S^{2}}}}$$

N represents the number of fluorescently labeled molecules in the FCS volume, τ_D_ is the characteristic diffusion correlation time, S denotes the structure parameter, T represents the fraction of fluorophores residing in the triplet state, and τ_T_ is the triplet lifetime. The hydrodynamic radius R_H_ of sample molecules was calculated from the diffusion correlation time τ_D_ using the following relationships:

$$\tau_{D}\;=\;\frac{r_{0}^{2}}{4D}\;\;\;\;and\;D\;=\;\frac{k_{B}T}{6\pi \eta R_{H}}$$

r_0_ represents the lateral dimension of the FCS volume, D is the diffusion coefficient, k_B_ is the Boltzmann constant, T is absolute experimental temperature, and η represents the sample buffer viscosity.

For titration experiments, varying concentrations of His-tagged, Nluc-fused nanobodies (G8, NB28, and NB26) were mixed with Cy5-labeled AFB1-BSA. To obtain the fractions of unbound and bound AFB1-BSA after incubation with G8, NB28, and NB26 fusion proteins, normalized autocorrelation curves were fit to a model of the diffusion of two species in a three-dimensional Gaussian confocal volume. All fitting was performed with a non-linear least-squares algorithm, and dissociation constants (KD) were determined by the Correlation Analysis software.

### 2.5. Structural Modelling and Molecular Docking

**Figure 1:**
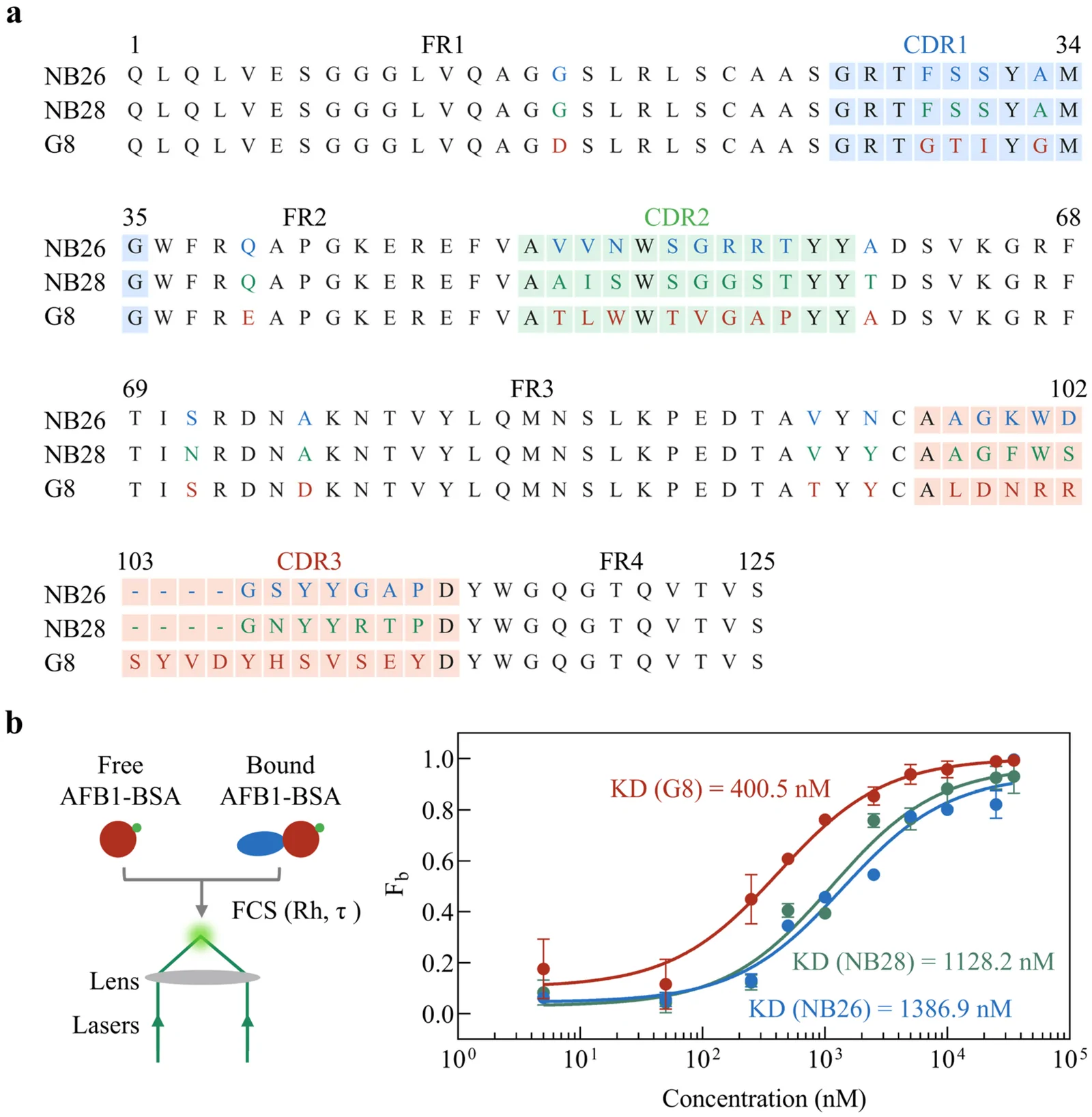
Sequence divergence and FCS affinity measurements identify G8 as the strongest AFB1-binding nanobody in a CDR3-divergent set. (**a**) Mature VHH-domain sequence alignment of NB26, NB28, and G8. CDR1, CDR2, and CDR3 are shaded blue, green, and red, respectively. Residue labels use mature display numbering; the initiating Met in the NB28 and NB26 structure sequences is omitted. Colored letters indicate non-identical aligned positions. The G8 CDR3 extension comprises aligned positions 103–106, which are gaps in NB28 and NB26. (**b**) FCS assay schematic (**left**) and concentration–response curves for AFB1-BSA binding to G8, NB28, and NB26 (**right**). The schematic depicts discrimination between free and nanobody-bound AFB1-BSA through changes in hydrodynamic radius (Rh) and diffusion time (tau). Bound fraction (Fb) is plotted against nanobody concentration on a logarithmic scale. Points show measured responses and solid lines show fitted binding curves.

The solution NMR structure of NB26 (PDB ID: 6IYN) was obtained from the RCSB Protein Data Bank [18,19]. Structural models of G8 and NB28 were generated in SWISS-MODEL using NB26 as the template [20]. The three-dimensional structure of AFB1 was retrieved from PubChem [21]. Protein-AFB1 docking was performed with HADDOCK [22]. Active residues for NB26 were defined from the AFB1-binding residues identified by NMR titration in the original NB26 structural study [14]: PHE37, PHE47, VAL50, TRP101, ASP102, GLY103, TYR105, TYR106, and PRO109 (mature display numbering). The residues at the corresponding aligned positions were used as active residues for G8 and NB28 (Figure 1a). The highest-scoring docked complex for each nanobody was selected as the starting structure for molecular dynamics (MD) simulation.

### 2.6. Molecular Dynamics Simulations

All AFB1-bound complexes were simulated with GROMACS 2020.2 [23]. Nanobodies were described with the CHARMM36 force field [24], and AFB1 parameters were generated with the CHARMM General Force Field (CGenFF) [25]. Each complex was placed in a cubic TIP3P water box with a minimum solute-to-box-edge distance of 1.0 nm [26]. Sodium and chloride ions were added to neutralize the systems and to obtain a final salt concentration of 0.15 M. Each solvated system was energy-minimized by the steepest-descent algorithm for a maximum of 50,000 steps, followed by 1 ns NVT and 1 ns NPT equilibration. Production simulations were performed in the NPT ensemble with a 2 fs integration step. Temperature was maintained at 298 K using the velocity-rescaling thermostat [27], and pressure was maintained at 1 bar using the isotropic Parrinello–Rahman barostat [28]. All bonds involving hydrogen atoms were constrained with LINCS [29]. Long-range electrostatic interactions were calculated using particle-mesh Ewald [30]; short-range Coulombic and van der Waals interactions used 1.2 nm cutoffs, with van der Waals switching initiated at 1.0 nm. Periodic boundary conditions were applied in all directions. Three independent 3 μs production trajectories were generated for each AFB1-bound nanobody complex, yielding nine trajectories and 27 μs of aggregate sampling.

### 2.7. Trajectory Processing and Global Dynamics Analyses

Trajectories were corrected for periodic boundary conditions, centered on the protein, and least-squares-fitted to remove global rotation and translation. Solvent-free trajectories were used for global dynamics, conformational clustering, and MM/GBSA calculations. Water-containing trajectories were retained for interaction-fingerprint analyses to detect water-mediated interactions. Protein C-alpha RMSD, AFB1 RMSD, radius of gyration (Rg), and per-residue C-alpha RMSF were calculated with MDAnalysis [31]. RMSD values were evaluated relative to the first trajectory frame after protein fitting. RMSF values were calculated after C-alpha alignment. For each system, time-series means and standard deviations were calculated across the three replicate trajectories; Rg distributions were additionally evaluated from all sampled frames.

### 2.8. Interaction-Fingerprint, Mapped-Contact, and Conformational Clustering Analyses

Protein–AFB1 interaction fingerprints (IFPs) were extracted from the water-containing trajectories using an MD-IFP workflow [32]. Aromatic (AR), hydrophobic (HY), hydrogen-bond donor (HD), hydrogen-bond acceptor (HA), and water-bridge (WB) contacts were assigned for each sampled frame. These are atom-level, geometry-based IFP classes; in particular, an ‘HY’ assignment denotes a nonpolar contact geometry and does not classify the whole amino-acid residue as intrinsically hydrophobic. Replicate-level contact occupancy was calculated as the fraction of frames containing the respective interaction. Contacts absent from a replicate were assigned zero occupancy before calculation of the three-replicate mean and standard deviation. Ring-edge contacts were excluded from the principal comparisons because their near-universal occurrence did not discriminate among the systems. For per-residue summaries, the best direct-contact or best AR/HY occupancy was defined as the highest mean occupancy among the corresponding IFP contact types assigned to that residue.

To compare corresponding contact regions, G8 reference residues were mapped to NB28 and NB26 through the aligned sequence. Direct-contact fractions and minimum distances between AFB1 and the mapped residues were calculated independently for each replicate. Cross-system occupancy matrices were indexed by aligned reference residue, whereas residue labels in text and figures use the mature display numbering of each nanobody. For conformational clustering, the three solvent-free replicate trajectories from each system were concatenated and clustered with the GROMOS algorithm in GROMACS using protein C-alpha RMSD after least-squares fitting. Cutoff sensitivity was assessed from 0.10 to 0.30 nm, and 0.18 nm was selected for the representative-structure analysis. The centers of the most populated cluster were used to visualize the IFP-defined contact sites.

### 2.9. MM/GBSA Calculations and Residue Decomposition

Binding energetics were estimated separately for each replicate using the single-trajectory MM/GBSA implementation in gmx_MMPBSA, with the nanobody designated as the receptor and AFB1 as the ligand [33]. The analysis used frames corresponding to 0.5–3.0 μs. Total binding-energy estimates and component terms were summarized as the mean and standard deviation across the three replicate trajectories. Per-residue decomposition was performed for the same sampling window. The total decomposition contribution (TDC) of each nanobody residue was averaged across replicates and compared with its best AR/HY IFP occupancy.

### 2.10. Joint Analysis of Contact Occupancy, Residue Decomposition, and Contact Co-Occurrence

Residues meeting both a best AR/HY IFP occupancy of at least 0.30 and a TDC no greater than −0.50 kcal⋅mol −1 were highlighted. Classification was performed at aligned residue positions, while node labels retained each nanobody’s mature display residue numbering. For each mapped position, a frame was classified as AR/HY-active when at least one aromatic or hydrophobic IFP contact was present. Pairwise contact co-occurrence was calculated as the fraction of analyzed frames in which both positions were AR/HY-active. Edges with co-occurrence of at least 0.25 were plotted as solid lines; edges with co-occurrence from 0.12 to less than 0.25 were plotted as dashed lines. Residues meeting both thresholds were distinguished from other residues participating in displayed contact pairs.

## 3. Results

### 3.1. G8 Has the Highest Affinity Among AFB1-Binding Nanobodies with Divergent CDR3 Sequences

To establish the observation that requires mechanistic explanation, we compared the mature VHH sequences of G8, NB28, and NB26 and quantified their binding to AFB1-BSA using FCS. The three nanobodies retain the characteristic VHH framework architecture but differ across all CDRs, most prominently in CDR3 (Figure 1a). G8 contains a four-residue CDR3 insertion at aligned positions 103–106 (SER103-TYR104-VAL105-ASP106) that is absent from NB28 and NB26. At aligned position 101, G8 encodes ARG101, whereas NB28 and NB26 both encode TRP101. This sequence relationship defines a comparative nanobody set with conserved framework architecture and distinct putative AFB1-binding surfaces.

Cy5-labeled AFB1-BSA produced nanobody concentration-dependent changes in the FCS autocorrelation curves for all three nanobodies (Figure S1). Fitting the derived binding responses yielded KD values of 400.5 nM for G8, 1128.2 nM for NB28, and 1386.9 nM for NB26 (Figure 1b). Accordingly, G8 displayed the strongest binding, NB28 displayed intermediate binding, and NB26 displayed the weakest binding under the assay conditions. The apparent KD of G8 was 2.8-fold lower than that of NB28 and 3.5-fold lower than that of NB26. This affinity order provides the experimental reference for comparative analysis of AFB1 interactions.

### 3.2. Stable Holo-State Trajectories Establish the Basis for Comparative Analysis of AFB1 Recognition

**Figure 2:**
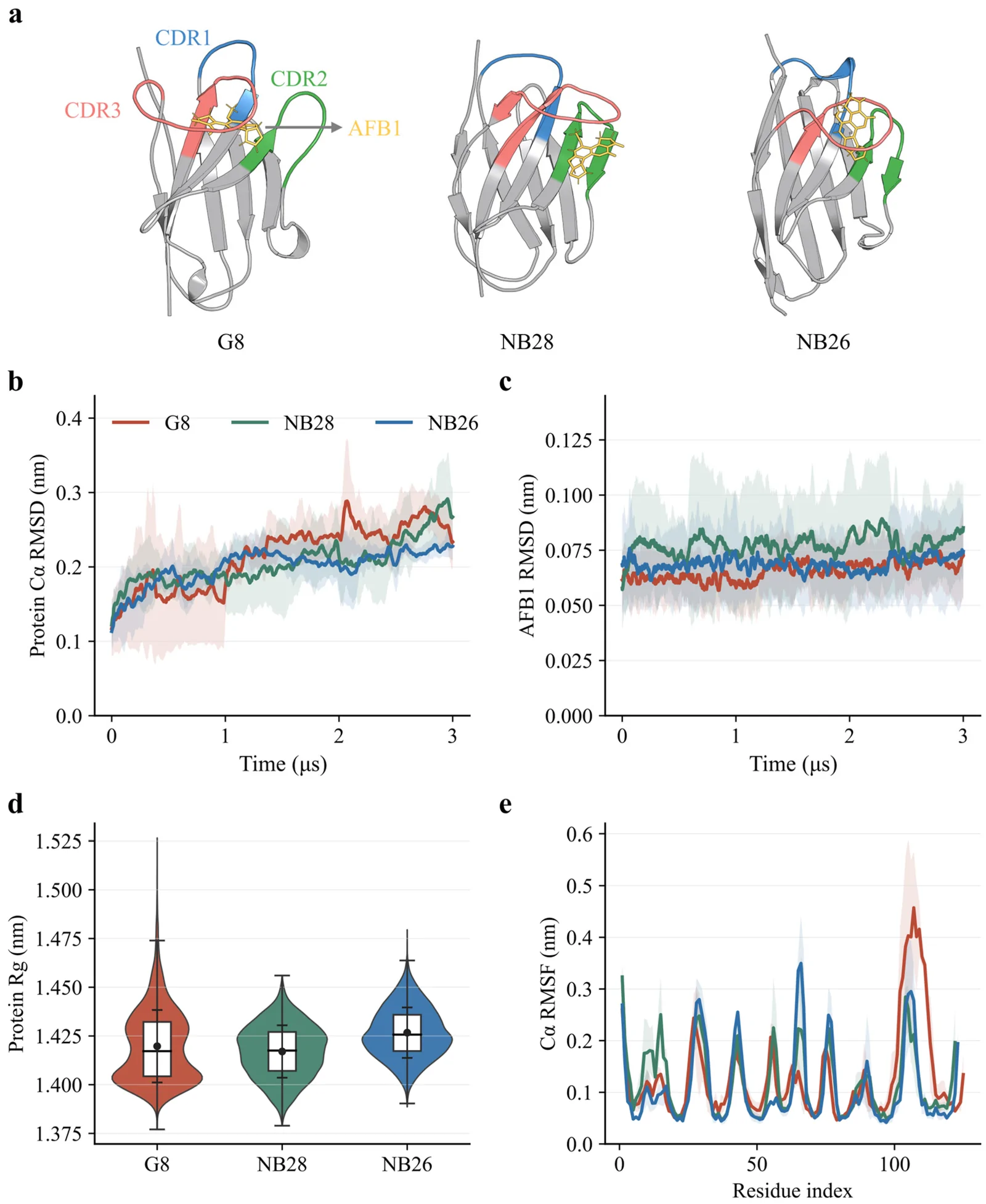
Comparative holo-state simulations yield stable AFB1-bound ensembles. (**a**) Initial structures of the G8-AFB1, NB28-AFB1, and NB26-AFB1 complexes used for molecular dynamics simulations. Nanobodies are shown as grey cartoons, with CDR1, CDR2, and CDR3 coloured blue, green, and salmon, respectively; AFB1 is shown as yellow sticks. (**b**) Protein C-alpha RMSD over three independent 3-μs simulations per nanobody–AFB1 complex. (**c**) AFB1 RMSD over the same trajectories. (**d**) Distribution of protein radius of gyration (Rg) across simulation frames. (**e**) Residue-wise C-alpha RMSF profiles. In (**b**,**c**,**e**), solid lines show the mean and shaded regions show the standard deviation across the three independent trajectories. In (**d**), violin plots show the frame-wise distribution, box plots show the interquartile range and median, and points with error bars show replicate means and their dispersion.

To interrogate the structural basis of the affinity separation, we generated comparable AFB1-bound models for the three nanobodies. The solution NMR structure of NB26 was used as the template for SWISS-MODEL homology modelling of NB28 and G8. AFB1 coordinates were obtained from PubChem. HADDOCK docking used the NB26 interface identified by NMR titration, together with the corresponding aligned positions in NB28 and G8 (Figure 1a), as restraints [14]. The highest-scoring pose for each nanobody was selected as the starting complex structure (Figure 2a).

Each complex was subjected to three independent 3 μs explicit-solvent molecular dynamics simulations. Protein C-alpha RMSD values reached stable plateaus after the initial relaxation phase and remained similar across systems, with trajectory means of 0.215 nm for G8, 0.206 nm for NB28, and 0.200 nm for NB26 (Figure 2b and Figure S2). AFB1 RMSD remained low in all three complexes (0.065, 0.078, and 0.069 nm for G8, NB28, and NB26, respectively), consistent with retention of the ligand near its initial binding site throughout the sampled trajectories (Figure 2c and Figure S2). Protein compactness was likewise comparable, with mean radii of gyration of 1.420, 1.417, and 1.427 nm, respectively (Figure 2d). Per-residue C-alpha RMSF identified localized loop and terminal mobility (Figure 2e). In G8, the major mobility peak spanned residues 102–112 within the extended CDR3 (residues 96–115) and reached 0.457 nm at TYR107. NB28 and NB26 displayed localized mobility near the CDR2-adjacent region, but this was not a uniform increase across the CDR2 loop. These global descriptors confirm complex stability across systems, while the distinct CDR3 flexibility in G8 pinpoints a local dynamic hotspot for subsequent analysis.

### 3.3. Interaction Fingerprint Analysis Identifies Distinct AFB1 Interaction Profiles

**Figure 3:**
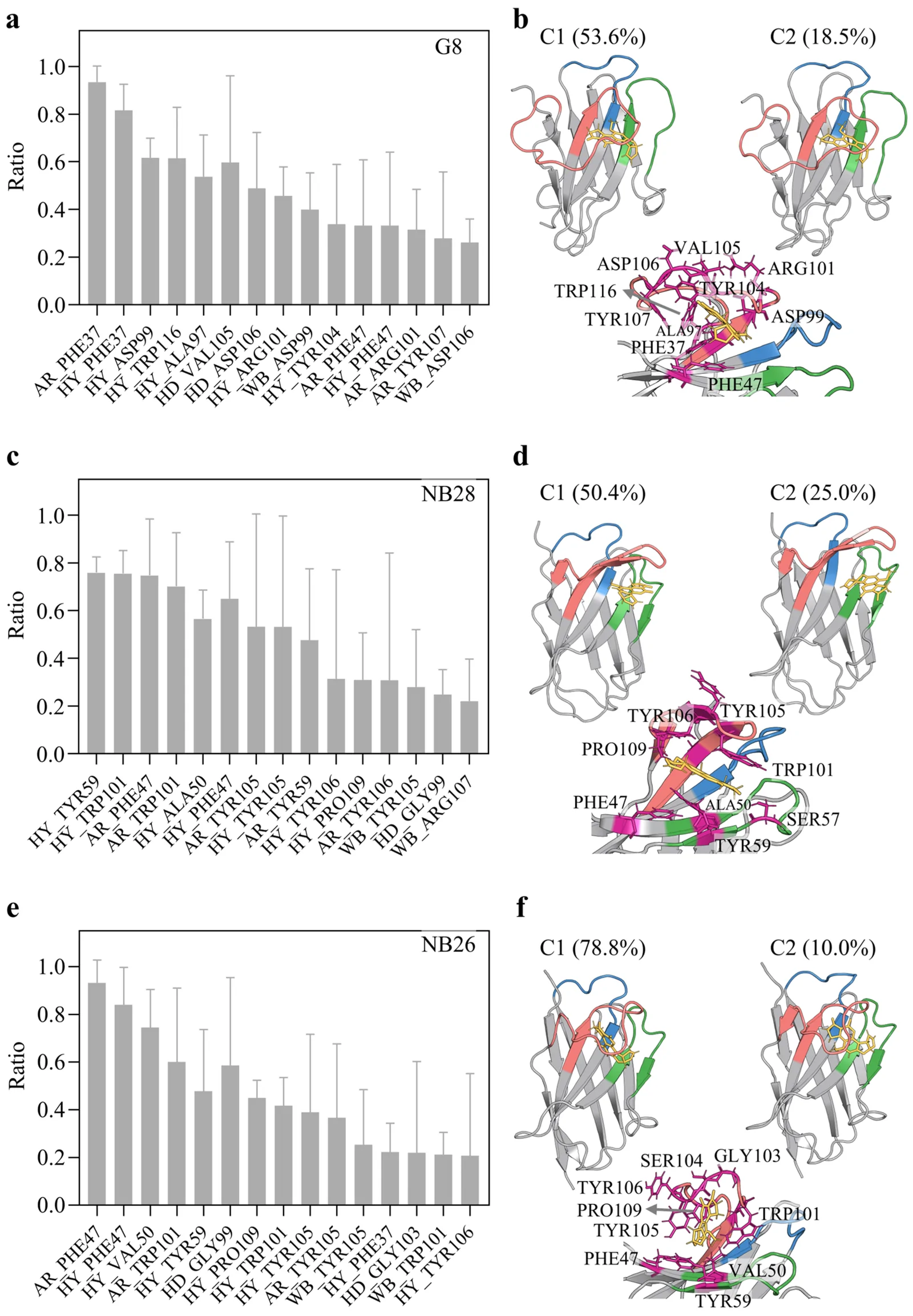
Dominant conformations and interaction-site landscapes of the three nanobody–AFB1 complexes. (**a**,**c**,**e**) The 15 most frequent AFB1 interaction fingerprint features for G8, NB28, and NB26, respectively. Bars show the mean occupancy across the three independent trajectories; error bars show the standard deviation. Interaction types are abbreviated as AR, aromatic; HY, hydrophobic; HD, hydrogen donor; and WB, water bridge. Residue labels use mature display numbering. (**b**,**d**,**f**) Cluster-center structures used to visualize the IFP-defined interaction sites in G8, NB28, and NB26, respectively. The two most populated clusters (C1 and C2) were obtained at a 0.18 nm RMSD cutoff; their percentages indicate the fraction of frames in the concatenated three-replicate ensemble. Upper panels show the C1 and C2 center structures. Lower panels show an enlarged view of the C1 representative. CDR1, CDR2, and CDR3 are colored blue, green, and salmon, respectively; AFB1 is shown in yellow, and the binding sites corresponding to the ranked interaction features are highlighted in magenta.

Apart from the CDR3 mobility in G8, the global metrics were largely similar across systems and did not correlate with the affinity order, prompting us to examine residue-level contacts. For each nanobody, the 15 most frequent IFP contacts, excluding ring-edge contacts, were ranked by zero-filled mean occupancy across the three independent trajectories; an undetected contact contributed zero occupancy for that replicate. All three complexes formed frequent aromatic and hydrophobic contacts at the CDR-facing binding interface, but these contacts were distributed across different residue sets (Figure 3a,c,e).

In G8, PHE37 gave the two highest-occupancy contacts, aromatic (0.934) and hydrophobic (0.816). PHE37 was accompanied by contacts across the G8 CDR3, including hydrophobic contacts at ALA97 (0.537), ASP99 (0.617), ARG101 (0.456), TYR104 (0.338), and TRP116 (0.615). For ASP99 and ARG101, the ‘HY’ designation reflects a geometric contact involving nonpolar atoms/portions of the residue and must not be interpreted as a classification of these polar residues as hydrophobic. In contrast, G8-specific VAL105 and ASP106 were observed mainly in hydrogen-donor and water-bridge features. For VAL105, these features are attributable to backbone- and/or water-mediated geometry rather than to a polar valine side chain. Thus, the G8 contact profile extended from PHE37 into the elongated CDR3. In contrast, the NB28 and NB26 profiles were centered on PHE47 and TRP101. In NB28, the leading contacts involved TYR59 (hydrophobic, 0.760), TRP101 (hydrophobic, 0.755; aromatic, 0.702), and PHE47 (aromatic, 0.748; hydrophobic, 0.649). In NB26, PHE47 dominated the profile (aromatic, 0.930; hydrophobic, 0.838), followed by a VAL50 hydrophobic contact (0.743) and a TRP101 aromatic contact (0.598). PHE37/PHE47 are located in FR2; VAL50 in NB26 and TYR59 in NB28 are located in CDR2; TRP101 and the other short-loop contact residues in NB26/NB28 are located in CDR3. In G8, ALA97, ASP99, ARG101, TYR104, VAL105, ASP106, and TYR107 are CDR3 residues, whereas TRP116 is located in FR4 immediately after CDR3 (Figure 1a). Thus, the three nanobodies shared aromatic and hydrophobic contact types but differed in their distribution across framework and CDR3 residues.

Trajectory clustering placed these IFP-defined contacts in representative complex conformations. A 0.18 nm RMSD cutoff was selected by cutoff-sensitivity analysis (Figure S3). The two most populated clusters accounted for 72.1% of G8, 75.4% of NB28, and 88.8% of NB26 frames; C1 alone accounted for 53.6%, 50.4%, and 78.8%, respectively (Figure 3b,d,f). NB26 was therefore concentrated in one dominant conformation, whereas G8 and NB28 populated two major conformations more evenly. Mapping IFP contact residues onto the C1 centers visualized a common CDR-facing AFB1–binding interface with distinct contact arrangements: G8 included PHE37, ALA97, ASP99, ARG101, and the extended CDR3, whereas NB28 and NB26 concentrated their leading contacts around PHE47, TRP101, and shorter CDR3 residues. These results define distinct AFB1 contact profiles across the three nanobodies.

### 3.4. Persistent AFB1 Contacts and Favorable Per-Residue MM/GBSA Contributions Distinguish G8 from NB28 and NB26

**Figure 4:**
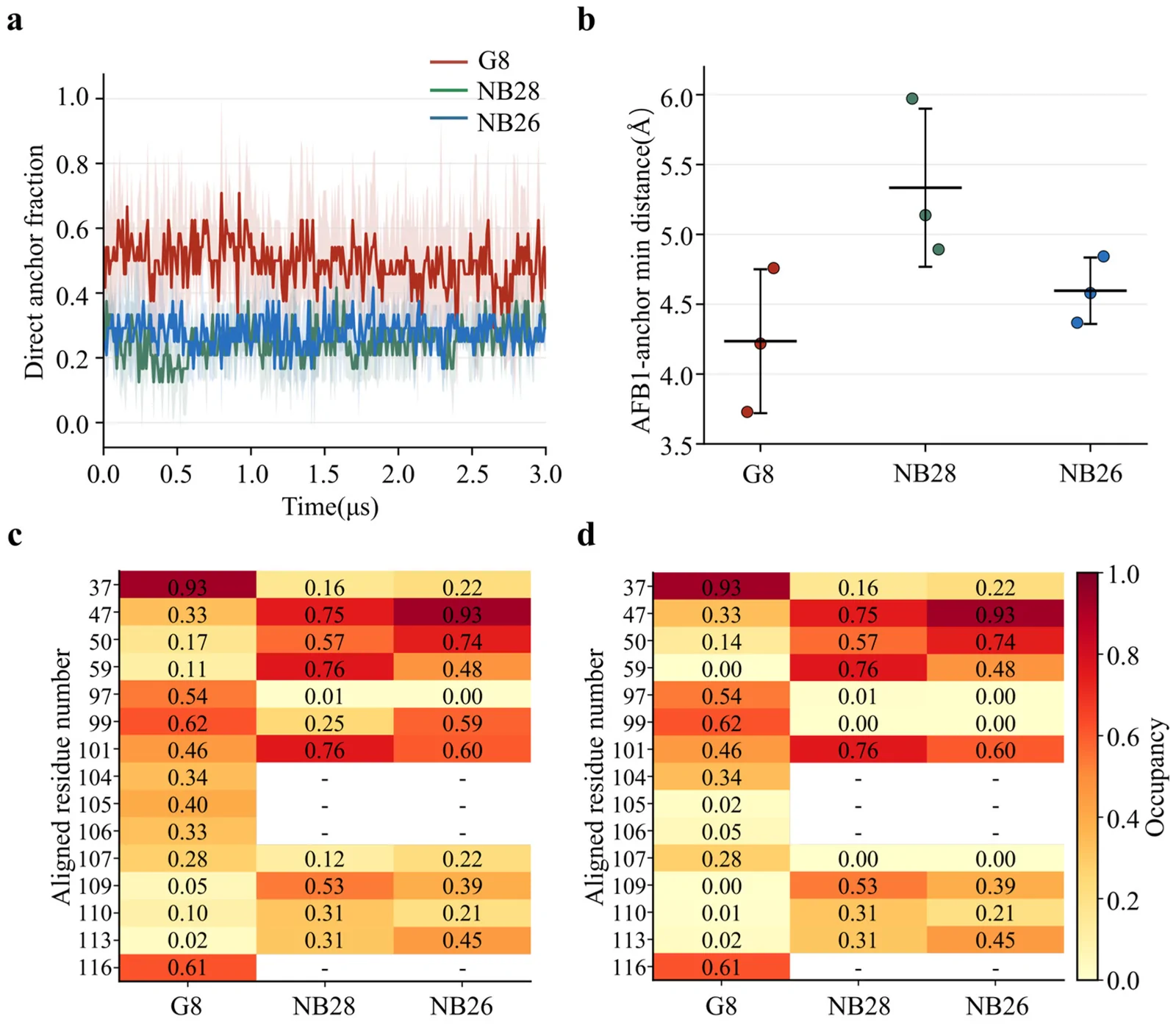
Persistent mapped contacts and aligned interaction fingerprints distinguish the AFB1 recognition networks. (**a**) Fraction of mapped spatial anchors forming direct AFB1 interactions over three independent 3 μs trajectories. Solid lines show the across-replicate mean and shaded regions show the standard deviation. (**b**) Mean minimum distance between AFB1 and the mapped anchor set for each replicate. Points denote replicate means; horizontal bars and error bars denote the system mean and standard deviation, respectively. (**c**) Best IFP occupancy at aligned contact positions. Each cell is the highest features assigned to that residue. Rows were included when at least one nanobody had an occupancy of at least 0.25. (**d**) Best aromatic/hydrophobic (AR/HY) IFP occupancy at the identical aligned positions and in the same order as panel (**c**).

The contact profiles in Figure 3 identify residues contacting AFB1; we next assessed the persistence of these contacts across the trajectories. The fraction of mapped reference residues in direct contact with AFB1 was higher for G8 (0.495) than for NB28 (0.248) or NB26 (0.277) throughout the simulations (Figure 4a). The mean minimum distance between AFB1 and the mapped residues was 4.24 Å for G8, 4.60 Å for NB26, and 5.33 Å for NB28 (Figure 4b). Although the mean value was lower for G8, the replicate-level variation overlaps with that of NB26; this comparison should therefore be interpreted descriptively rather than as a resolved difference between these two systems. In contrast, the direct-contact fraction was higher for G8 than for NB28 or NB26, supporting a broader set of persistent mapped contacts in G8.

The aligned IFP profiles show the contribution of individual residues to these contacts (Figure 4c,d). G8 maintained aromatic/hydrophobic contacts at PHE37 (0.934), ALA97 (0.537), ASP99 (0.617), ARG101 (0.456), TYR104 (0.338), TYR107 (0.278), and TRP116 (0.615). In contrast, NB28 and NB26 had high occupancies at PHE47, aligned positions 50 and 59, and TRP101; the TRP101 occupancies were 0.755 and 0.598, respectively. G8 did not have the highest occupancy at every aligned position. Rather, aromatic/hydrophobic contacts extended from the conserved PHE37/PHE47-equivalent position to ALA97, ARG101, the G8-specific TYR104 insertion position, and TRP116. VAL105 and ASP106 also had direct-contact occupancies of 0.398 and 0.326, respectively, but little aromatic/hydrophobic occupancy, consistent with polar or water-mediated contacts rather than aromatic or hydrophobic contacts.

**Figure 5:**
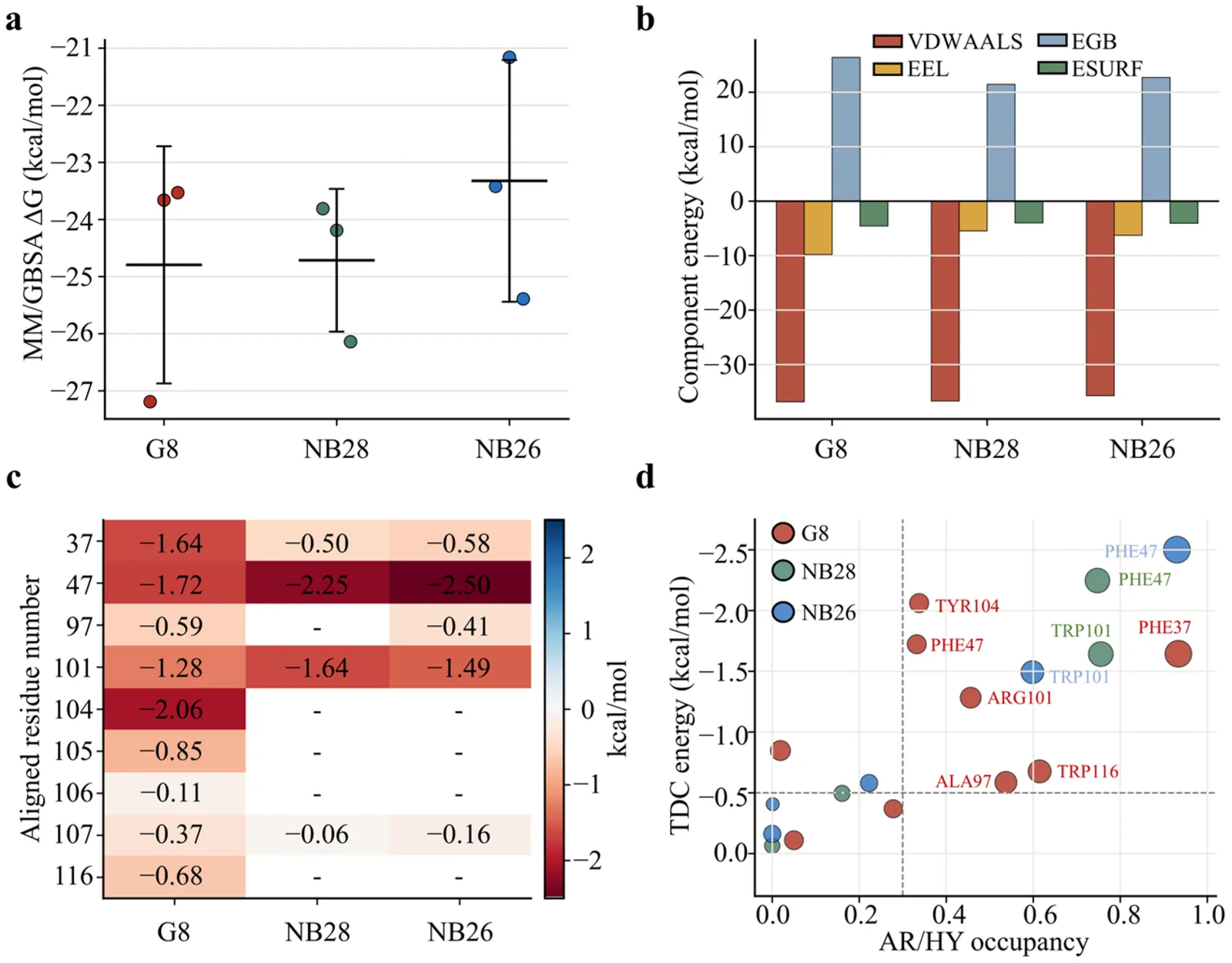
Residue-level MM/GBSA decomposition identifies energetic support for persistent recognition sites. (**a**) Replicate-level MM/GBSA total binding free energies (ΔG) for the three complexes. Points represent independent trajectories; horizontal bars and error bars show the mean and standard deviation. (**b**) Mean MM/GBSA energy components: van der Waals (VDWAALS), electrostatic (EEL), polar solvation (EGB), and nonpolar solvation (ESURF). (**c**) TDC residue-decomposition values at selected aligned positions. Negative values indicate favorable residue-level contributions; dashes indicate that a corresponding residue or decomposition value is unavailable. (**d**) Integration of residue-level best AR/HY IFP occupancy and TDC for the selected positions. Dashed lines indicate the EPM thresholds of occupancy ≥ 0.30 and TDC ≤ −0.50 kcal⋅mol^−1^.

We next assessed whether residues with persistent contact also had favorable per-residue MM/GBSA contributions. MM/GBSA total binding energies were favorable for all three complexes, with means of −24.79, −24.71, and −23.32 kcal⋅mol^−1^ for G8, NB28, and NB26, respectively (Figure 5a). Favorable van der Waals contributions were partially offset by polar solvation in each system (Figure 5b). The similar G8 and NB28 total values indicate that ΔG does not quantitatively account for the FCS affinity order. In contrast, total decomposition contribution (TDC) values were favorable for G8 PHE37 (−1.64 kcal⋅mol^−1^), PHE47 (−1.72 kcal⋅mol^−1^), ALA97 (−0.59 kcal⋅mol^−1^), ARG101 (−1.28 kcal⋅mol^−1^), TYR104 (−2.06 kcal⋅mol^−1^), and TRP116 (−0.68 kcal⋅mol^−1^) (Figure 5c). The contact-occupancy and decomposition comparison also identified PHE47 and TRP101 with persistent aromatic/hydrophobic contacts and favorable TDC values in NB28 and NB26 (Figure 5d). Thus, the broader set of persistent G8 contacts is supported by favorable per-residue contributions, rather than by a uniquely favorable total MM/GBSA value or a single dominant contact.

### 3.5. Joint Analysis of Contact Occupancy and Residue Decomposition Unveils Distinct Recognition Architectures Among AFB1-Binding Nanobodies

**Figure 6:**
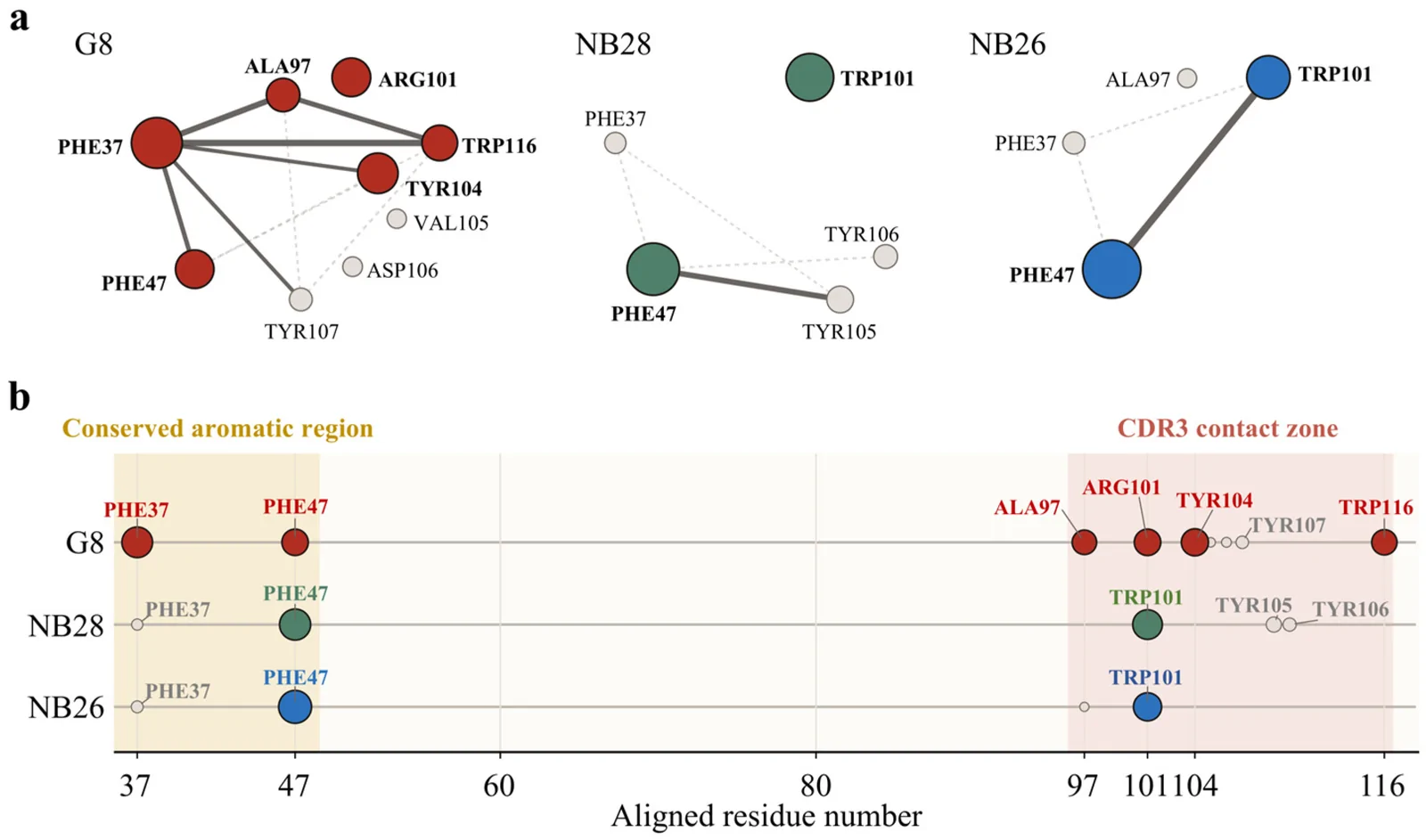
Joint analysis of contact occupancy, per-residue MM/GBSA decomposition, and contact co-occurrence identifies distinct AFB1 contact profiles. (**a**) Dynamic AR/HY co-occupancy networks for G8, NB28, and NB26. Colored nodes are EPM residues, defined by best AR/HY occupancy ≥ 0.30 and TDC ≤ −0.50 kcal⋅mol^−1^. Grey nodes are non-EPM residues participating in a displayed edge. Solid dark edges denote strong co-occupancy (≥0.25), whereas dashed grey edges denote weaker displayed co-occupancy (0.12 to <0.25). (**b**) Aligned position map of the displayed network residues. Labels use each nanobody’s mature display numbering; the beige region marks the conserved aromatic base, and the rose region marks the CDR3 recognition zone.

The preceding analyses identify persistent contacts and favorable TDC values, but neither descriptor alone indicates whether these residues contact AFB1 simultaneously. In Figure 6, residues with best AR/HY occupancy of at least 0.30 and TDC no greater than −0.50 kcal⋅mol^−1^ are highlighted. These thresholds provide an operational display of two descriptors and do not represent a separate physical quantity. Frame-resolved pairwise AR/HY contact co-occurrence was then calculated to identify residues with concurrent AFB1 contacts (Figure 6a).

G8 contained six residues meeting both thresholds, PHE37, PHE47, ALA97, ARG101, TYR104, and TRP116, and six residue pairs with AR/HY contact co-occurrence of at least 0.25. These residues spanned the conserved aromatic contact region and G8-specific CDR3 positions at the AFB1-binding interface. In contrast, NB28 and NB26 each contained two residues meeting both thresholds, PHE47 and TRP101, and one residue pair with co-occurrence of at least 0.25. In NB26, this pair was PHE47-TRP101; in NB28, PHE47 co-occurred with TYR105, which did not meet both thresholds. The aligned-position map therefore shows a broader set of concurrently persistent G8 contacts than the more localized contact profiles of NB28 and NB26 (Figure 6b).

The comparison provides a basis for prioritizing residues in AFB1-binding nanobody engineering. Conserved aromatic residues may provide baseline ligand contacts, whereas variable CDR3 residues warrant particular attention when they make persistent contacts, have favorable per-residue MM/GBSA contributions, and co-occur with other AFB1 contacts. The TYR104-containing G8 region illustrates this criterion because its selection is supported by contact occupancy, TDC, and contact co-occurrence rather than by one descriptor alone (Figure 6).

## 4. Discussion

The main finding indicates that the differences in affinity among G8, NB28, and NB26 are associated with varying patterns of AFB1 contacts rather than with significant differences in the overall stability of the complexes. G8 demonstrated the lowest dissociation constant (KD), while parameters such as protein RMSD, AFB1 RMSD, and Rg remained similar across the three simulated complexes (see Figure 1 and Figure 2). Conversely, contact-level analyses revealed that G8 maintained aromatic and hydrophobic interactions from conserved residues into CDR3, whereas NB28 and NB26 were predominantly characterized by contacts around PHE47 and TRP101 (see Figure 3 and Figure 4). This distinction is important because, although global trajectory descriptors facilitate comparison of the simulations, they do not, in isolation, elucidate a molecular basis for the observed affinity hierarchy.

The present comparison extends the established AFB1-binding model for NB26. NMR titration and alanine-scanning experiments previously localized the NB26-AFB1 binding interface principally to FR2 and CDR3, identified important hydrophobic and hydrogen-bonding interactions, and showed that FR2/CDR3 substitutions could improve NB28 assay sensitivity [14]. Our simulations are consistent with this multi-region binding interface but further resolve its variation within a related nanobody set. PHE47 and TRP101 remain prominent contact residues in NB28 and NB26, whereas G8 includes ALA97, ARG101, TYR104, and TRP116 in addition to conserved aromatic positions (Figure 3 and Figure 4). The G8-specific 103–106 insertion should therefore not be treated as a uniform affinity motif: TYR104 and ARG101 made recurrent aromatic/hydrophobic contacts with favorable TDC values, whereas VAL105 and ASP106 primarily contributed direct polar or water-mediated contacts (Figure 4 and Figure 5).

The combined contact and energy analyses provide convergent, but distinct, evidence for a multiresidue contribution to G8 binding. The total MM/GBSA values for G8 and NB28 were similar and did not reproduce the experimental KD order (Figure 5a,b). This result is consistent with the recognized limitations of endpoint MM/PBSA and MM/GBSA calculations, which do not fully account for conformational entropy and solvent reorganization in experimental binding free energies [34]. In contrast, the per-residue decomposition identified favorable contributions from persistent G8 contact residues, including PHE37, PHE47, ALA97, ARG101, TYR104, and TRP116 (Figure 5c,d). The pairwise contact analysis further showed that these residues participate in a larger set of concurrently formed contacts in G8 than in NB28 or NB26 (Figure 6). Thus, the data supports a distributed contact model for G8 rather than an interpretation based on one residue or on total MM/GBSA alone.

This analysis suggests a practical route for structure-guided optimization of small-molecule-binding nanobodies. Candidate substitutions could be prioritized when they are predicted to preserve or introduce recurrent ligand contacts at multiple residues, particularly when those residues also show favorable per-residue decomposition contributions. This principle is compatible with the successful FR2/CDR3-guided modification of NB28 in the earlier AFB1 study [14] and with recent nanobody engineering work in which interaction-fingerprint analysis was used to select residues for subsequent experimental testing [35]. In the present set, TYR104, ARG101, and TRP116 are the most direct candidates for mutagenesis because the first is unique to the G8 insertion and all three contribute to the G8 contact pattern (Figure 4, Figure 5 and Figure 6). Their effects must nevertheless be established by direct binding measurements, because the energetic and structural consequence of a substitution depends on its sequence and local structural context.

More broadly, this integrative analysis offers a practical framework for identifying cooperative binding networks in small-molecule nanobody engineering. While conventional contact maps only capture static spatial proximity, incorporating pairwise co-occurrence distinguishes synchronized, cooperative contact clusters from isolated residues. This distinction is particularly relevant for targeting rigid haptens, where binding typically relies on concerted aromatic stacking and shape complementarity [36]. When applied to comparative datasets of related VHHs, this workflow systematically translates molecular dynamics trajectories into prioritized, testable hypotheses for engineering high-affinity detection reagents in food safety.

## 5. Conclusions

Comparative analyses of FCS and molecular dynamics simulations suggest that the enhanced binding of G8 to AFB1 is associated with a broader set of persistent contacts, rather than reliance on a single dominant interaction or a unique global-stability signature. Throughout the bound complexes, G8 exhibits persistent aromatic and hydrophobic contacts, complemented by favorable per-residue MM/GBSA contributions at conserved residues and G8-specific CDR3 positions. The integrated analysis of contact occupancy, residue decomposition, and contact co-occurrence further differentiates the G8 contact profile from the more localized profiles centered on PHE47 and TRP101 in NB28 and NB26. These results support using contact occupancy and per-residue MM/GBSA decomposition to interpret affinity differences among related nanobodies and prioritize mutations in small-molecule-binding nanobodies suitable for experimental validation.

## Data Availability

The original contributions presented in this study are included in the article/Supplementary Materials. Further inquiries can be directed to the corresponding author.

## Supplementary Materials

The following supporting information can be downloaded at https://www.mdpi.com/article/10.3390/biom16091265/s1. Figure S1: Fluorescence correlation spectroscopy (FCS) analysis of the binding of G8, NB26, and NB28 to AFB1-BSA; Figure S2: Analysis of clustering sensitivity for molecular dynamics trajectory data; Figure S3: Cluster-cutoff sensitivity supports selection of a 0.18 nm RMSD cutoff for holo-state representative structures.

## Abbreviations

The following abbreviations are used in this manuscript:

AFB1	Aflatoxin B1
VHHs	Variable domains of camelid heavy-chain-only antibodies
CDR	Complementarity-determining region
FR	Framework region
FCS	Fluorescence correlation spectroscopy
MD	Molecular dynamics
Nluc	NanoLuc luciferase
KD	Dissociation constants
Rg	Radius of gyration
IFPs	Interaction fingerprints
AR	Aromatic
HY	Hydrophobic
HD	Hydrogen-bond donor
HA	Hydrogen-bond acceptor
WB	Water-brodge
TDC	Total decomposition contribution
Rh	Hydrodynamic radius
Tau	Diffusion time
Fb	Bound fraction
ΔG	Total binding free energies
EEL	Electrostatic
EGB	Polar solvation
EPM	Energetically Pertinent Motif
NMR	Nuclear Magnetic Resonance
RMSD	Root Mean Square Deviation
RMSF	Root Mean Square Fluctuation
Cy5	sulfo-Cyanine5 NHS ester
CGenFF	CHARMM General Force Field
AFB1-BSA	Aflatoxin B1-Bovine Serum Albumin conjugate
MM/GBSA	Molecular Mechanics/Generalized Born Surface Area
ESURF	Nonpolar solvation
VDWAALS	van der Waals
