# Supplementary material for: Contact Occupancy and Per-Residue Energetic Contributions Distinguish Aflatoxin B1 Nanobodies with Different Binding Affinities
Source: Biomolecules. 2026 Sep 1;16(9):1265. doi: 10.3390/biom16091265 (PMC13604050; doi:10.3390/biom16091265)
Supplement: Supplementary file 1 [file biomolecules-16-01265-s001.zip › biomolecules-4520778-supplementary.pdf]

Supporting Information

Content

Fig. S1. Fluorescence correlation spectroscopy (FCS) analysis of the binding of G8, NB26, and NB28 to AFB1-BSA.

Fig. S2. Analysis of clustering sensitivity for molecular dynamics trajectory data.

Fig. S3. Cluster-cutoff sensitivity supports selection of a 0.18-nm RMSD cutoff for holo-state representative structures.

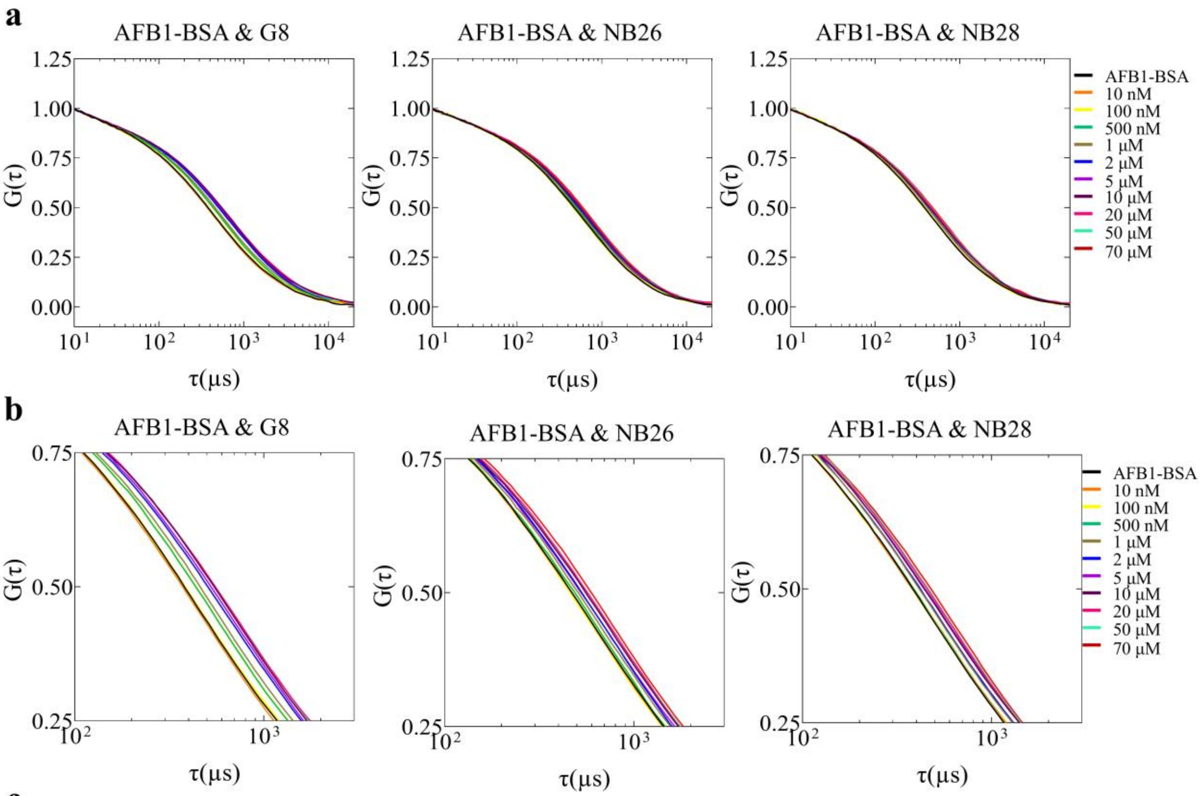

c

	G8		NB26		NB28	
**Conc.**	**τD (ms)**	**Fb**	**τD (ms)**	**Fb**	**τD (ms)**	**Fb**
0 nM	0.4299	0.0000	0.5383	0.0000	0.4253	0.0000
10 nM	0.4172	0.1754	0.5324	0.0633	0.4280	0.0835
100 nM	0.4388	0.1158	0.5358	0.0563	0.4240	0.0435
500 nM	0.4928	0.4490	0.5547	0.1313	0.4344	0.1229
1 μM	0.5308	0.6073	0.5856	0.3452	0.4653	0.4053
2 μM	0.5828	0.7608	0.6098	0.4571	0.4702	0.3941
5 μM	0.6046	0.8533	0.6219	0.5458	0.5074	0.7578
10 μM	0.6390	0.9376	0.6543	0.7748	0.5060	0.7647
20 μM	0.6528	0.9577	0.6640	0.8008	0.5164	0.8821
50 μM	0.6446	0.9884	0.6749	0.8210	0.5254	0.9248
70 μM	0.6490	0.9925	0.6956	0.9957	0.5401	0.9302

Fig. S1. Fluorescence correlation spectroscopy (FCS) analysis of the binding of G8, NB26, and NB28 to AFB1-BSA. (a) Normalized autocorrelation functions for AFB1-BSA alone (black) and after addition of the indicated nanobody concentrations (10 nM to 70 uM). (b) Local enlargement of the titration curves. (c) The fitted diffusion-correlation times (τD) and bound fractions (Fb).

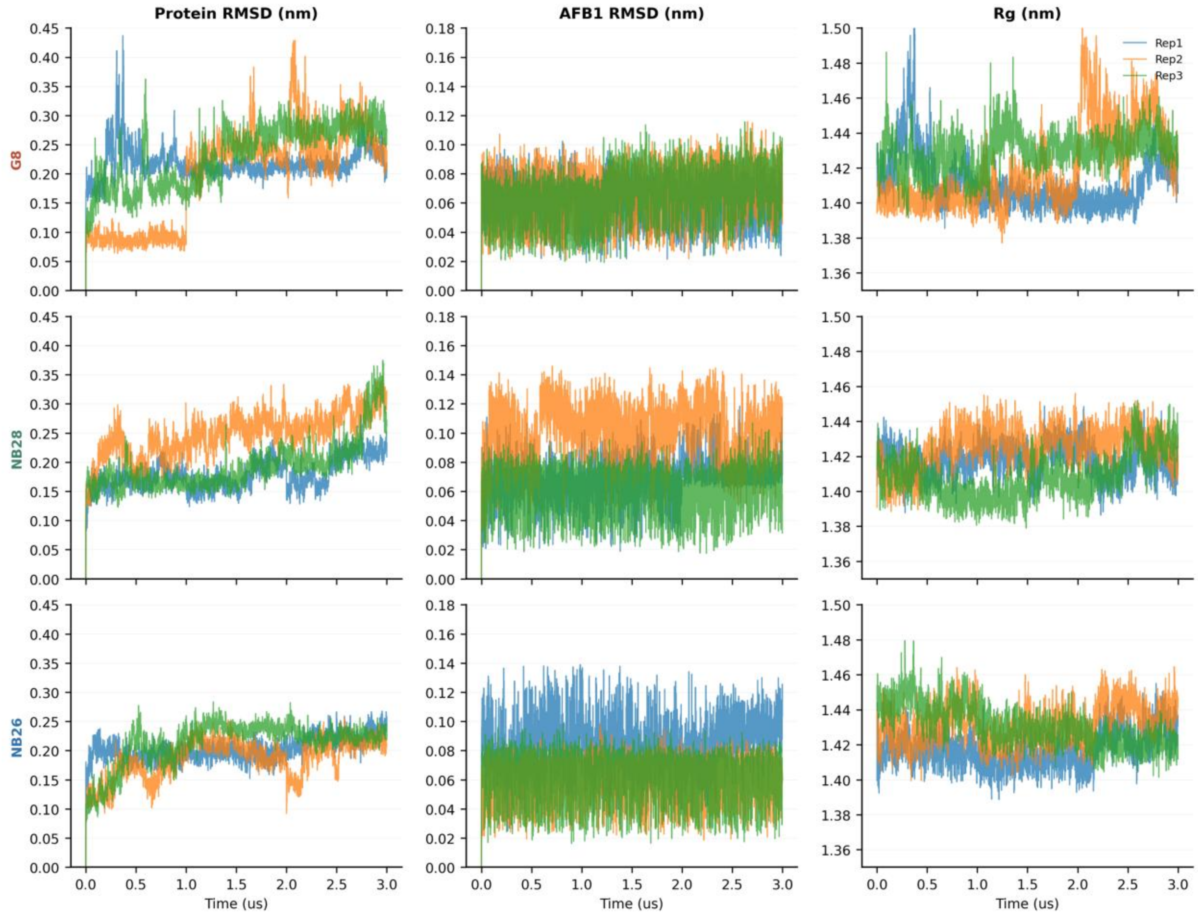

Fig. S2. Analysis of clustering sensitivity for molecular dynamics trajectory data. Protein RMSD, AFB1 RMSD, and protein radius of gyration (Rg) are shown over 3 μs for each independent trajectory. Rows show G8, NB28, and NB26 complexes; columns show protein RMSD, AFB1 RMSD, and Rg, respectively. Replicate 1, replicate 2, and replicate 3 are shown in blue, orange, and green.

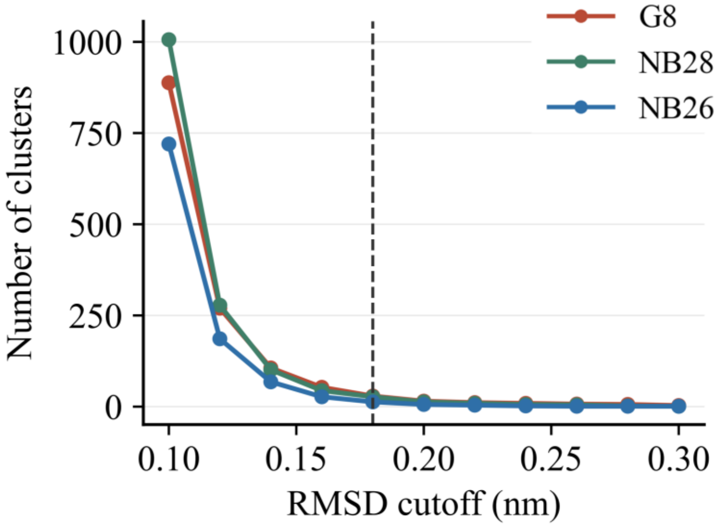

Fig. S3. Cluster-cutoff sensitivity supports selection of a 0.18-nm RMSD cutoff for holo-state representative structures. Number of clusters obtained from the concatenated G8, NB28, and NB26 trajectories as a function of the RMSD cutoff. The dashed vertical line marks the 0.18-nm cutoff.
